# Comparative In Vitro Resistance of Human Periodontal Bacterial Pathogens to Tinidazole and Four Other Antibiotics

**DOI:** 10.3390/antibiotics9020068

**Published:** 2020-02-07

**Authors:** Thomas E. Rams, Jacqueline D. Sautter, Arie J. van Winkelhoff

**Affiliations:** 1 Department of Periodontology and Oral Implantology, Temple University School of Dentistry, Philadelphia, PA 19140, USA; 2 Department of Microbiology and Immunology, Temple University School of Medicine, Philadelphia, PA 19140, USA; 3 Department of Medical Microbiology, University of Groningen, University Medical Center Groningen, 9713 GZ Groningen, The Netherlands; 4 Department of Periodontology, University of Groningen, University Medical Center Groningen, Center for Dentistry and Oral Hygiene, 9713 GZ Groningen, The Netherlands

**Keywords:** anti-infective agents, periodontitis, drug resistance, in vitro, periodontal pocket, tinidazole, metronidazole, doxycycline, amoxicillin, clindamycin

## Abstract

The in vitro resistance of selected red/orange complex periodontal pathogens to tinidazole was compared with four other antibiotics. Subgingival biofilm samples from 88 adults with severe periodontitis were anaerobically incubated on enriched Brucella blood agar with and without supplementation with tinidazole (16 mg/L), metronidazole (16 mg/L), amoxicillin (8 mg/L), doxycycline (4 mg/L), or clindamycin (4 mg/L). Growth of *Porphyromonas gingivalis, Tannerella forsythia, Prevotella intermedia/nigrescens, Parvimonas micra, Fusobacterium nucleatum*, *Streptococcus constellatus*, or *Campylobacter rectus* on antibiotic-supplemented plates indicated their in vitro antibiotic resistance. Tinidazole inhibited all test species, except *P. intermedia/nigrescens*, *P. micra*, and *S. constellatus* in 3.8%, 10.2%, and 88.9% of species-positive patients, respectively. Significantly fewer patients yielded tinidazole-resistant test species, and had significantly lower subgingival proportions of tinidazole-resistant organisms, than patients with amoxicillin, doxycycline, or clindamycin-resistant species, but not those with metronidazole-resistant strains. Joint in vitro species resistance to tinidazole and amoxicillin, or metronidazole and amoxicillin, was rare. Tinidazole performed in vitro similar to metronidazole, and markedly better than amoxicillin, doxycycline, or clindamycin, against fresh clinical isolates of red/orange complex periodontal pathogens. As a result of its similar antimicrobial spectrum, and more convenient once-a-day oral dosing, tinidazole should be considered in place of metronidazole for systemic periodontitis drug therapy.

## 1. Introduction

Human periodontitis is a destructive form of periodontal disease that is triggered by pathogenic bacterial biofilms, possibly in synergy with certain lytic herpesviruses [1], and mediated by dysregulated host hyper-inflammatory responses, causing progressive connective tissue attachment loss and alveolar bone resorption around teeth, ultimately leading to their loss from the oral cavity [2]. Among the approximately 700 known microbial species and uncultivated phylotypes that inhabit the human oral cavity, only a subset is associated with a pathogenic subgingival microbial dysbiosis in periodontitis-affected patients [3]. Socransky et al. [4], using DNA hybridization data, identified several clusters of bacteria as significantly related to various periodontal clinical conditions, with species belonging to the red and orange complex clusters most strongly associated with severe forms of periodontitis. The same red/orange complex species were also identified as part of the core subgingival microbiome of severe human periodontitis lesions in more recent studies using next generation gene sequencing [5,6]. Importantly, all red complex (*Porphyromonas gingivalis*, *Tannerella forsythia*, and *Treponema denticola*), and most orange complex species (including *Prevotella intermedia*, *Prevotella nigrescens*, *Parvimonas micra*, and *Fusobacterium nucleatum* group species, but not *Streptococcus constellatus* or *Campylobacter rectus*), are obligate anaerobic microorganisms, indicative of a strong association that exists between specific anaerobic bacteria and severe periodontitis [7].

Related to this, oral administration of metronidazole, which is highly active against anaerobic bacteria, is beneficial in enhancing therapeutic outcomes in severe periodontitis patients beyond that attained by conventional mechanically-based forms of periodontal therapy alone [8,9,10,11,12,13], due in large part to a significantly greater suppression of subgingival red/orange complex species achieved with systemic metronidazole therapy [14,15,16,17].

However, patient compliance with taking systemic metronidazole, which is most often prescribed to be taken orally three times per day for 7–14 days [18,19,20], is critical to the drug’s potential value in periodontal therapy. In a clinical trial, only 56% of the study patients were considered adequately compliant with taking systemic metronidazole tablets at the prescribed frequency of three times per day as an adjunct to conventional periodontal root debridement therapy, with compliant patients experiencing significantly better periodontal treatment outcomes that were more than double in magnitude as found in less compliant patients [21].

Tinidazole, a second-generation 2-methyl-5-nitroimidazole class antibiotic structurally similar to metronidazole, which exerts marked bactericidal activity against anaerobic bacteria and protozoa [22], may help overcome these patient compliance problems, and serve as a potential alternative to metronidazole and other antibiotics in combatting periodontal infections. Tinidazole possesses pharmacokinetic properties which enables once-a-day oral systemic drug dosing [23], a medication frequency associated with markedly better patient consumption compliance [24]. However, tinidazole, which was first clinically employed in 1969 for *Trichomonas vaginalis* infections [25], and approved for use in the United States by the Food and Drug Administration in 2004 [26], has to date received relatively little research attention for its potential administration in human periodontal disease therapy [27]. Systemic tinidazole has shown efficacy in treatment of pericoronitis lesions and acute periodontal abscesses [28], was reported to provide a subjectively higher effective treatment rate in periodontitis patients than systemic metronidazole (73.1% versus 43.5%) [29], and in smoking periodontitis patients, induced significantly more favorable changes in probing depth, clinical periodontal attachment level, and gingival inflammation than conventional periodontal root scaling alone [30]. Only limited data are presently available on the antimicrobial effects of tinidazole on various putative human periodontal bacterial pathogens [31,32], particularly in comparison to other potential systemic periodontal antibiotic drug choices [18,19,20].

To further advance consideration of tinidazole in periodontal disease management, this study determined and compared the prevalence and levels of in vitro resistance among fresh clinical isolates of selected red/orange complex bacterial species from severe periodontitis patients to tinidazole and four other antibiotics often systemically employed in human periodontal disease therapy.

## 2. Materials and Methods

### 2.1. Microbial Specimens

Subgingival plaque biofilm specimens were used from 88 systemically healthy, non-smoking adults (34 male, 54 female; mean age = 57.2 ± 13.3 (standard deviation, SD) years; age range = 35–83 years old) with severe periodontitis from whom subgingival samples were consecutively submitted by USA private practicing periodontists for microbiological analysis and antibiotic resistance testing to metronidazole, amoxicillin, doxycycline, and clindamycin at the Oral Microbiology Testing Service (OMTS) Laboratory at Temple University School of Dentistry, Philadelphia, PA, USA, which is licensed for high complexity bacteriological analysis by the Pennsylvania Department of Health. Samples from patients identified with aggressive periodontitis or antibiotic use 6 months before sampling were excluded. The subgingival specimens, which are normally discarded by the OMTS Laboratory after completion of requested microbiological testing, were additionally used in this study for tinidazole in vitro resistance testing after removal of all unique patient identifiers.

### 2.2. Microbial Sampling and Transport

The subgingival specimens were obtained with sterile paper points by the treating periodontists from 3–5 periodontal sites exhibiting deep periodontal probing depths (≥7 mm) and bleeding on probing, following standardized sampling procedures previously described [33]. All paper points per patient were pooled into a single glass vial containing pre-reduced, anaerobically sterilized and stored Möller’s VMGA III transport medium [34], and delivered within 24 hours to the OMTS Laboratory.

### 2.3. Microbial Culture

The microbial specimens were processed as previously described [33], with aliquots of serial 10-fold dilutions inoculated onto enriched Brucella blood agar (EBBA) plates with either no antibiotics added, or supplemented with non-susceptible breakpoint concentrations of either tinidazole (16 mg/L), metronidazole (16 mg/L), amoxicillin (8 mg/L), doxycycline (4 mg/L), or clindamycin (4 mg/L) (all antibiotics obtained as pure powder from Sigma-Aldrich, St. Louis, MO, USA). The breakpoint concentration values for metronidazole, amoxicillin, doxycycline, and clindamycin were employed in previous periodontal and dental implant microbiology studies [33,35], and represent drug levels at or above minimal inhibitory concentrations associated with the “susceptible” interpretative category for anaerobic bacteria as recognized by the Clinical and Laboratory Standards Institute (CLSI) [36], or the French Society for Microbiology (for doxycycline) [37]. The 16 mg/L non-susceptible breakpoint concentration for metronidazole was also used for tinidazole in this study, since breakpoint concentrations for tinidazole are not established, but are considered to be equivalent to those for metronidazole [32]. All inoculated EBBA plates were incubated at 37 °C for 7 days in a upright heated incubator (Caron, Marietta, OH, USA) in jars containing an 85% N_2_-10% H_2_-5% CO_2_ anaerobic atmosphere introduced by an automatic jar evacuation-replacement system (Anoxomat Mark II, Advanced Instruments, Inc., Norwood, MA, USA).

### 2.4. Microbial Identification

After incubation, established phenotypic criteria [33] were used to quantitate total anaerobic viable counts, and determine the presence and proportional recovery of selected red/orange complex species, including *P. gingivalis, T. forsythia, P. intermedia/nigrescens, P. micra, F. nucleatum* group species, *S. constellatus*, and *C. rectus*.

In brief, *P. gingivalis* was identified as circular, dome-shaped, dark-pigmented (brown to tan), raised surfaces colonies that lacked brick-red autofluorescence under long-wave ultraviolet light, but exhibited trypsin-like enzyme activity. *T. forsythia* was identified as Gram-negative, non-motile, anaerobic rods exhibiting gray-pink speckled, convex, pinpoint colonies seen with a stereomicroscope, lack of long-wave ultraviolet light autofluorescence, and positive for trypsin-like enzyme activity. *P. intermedia/nigrescens* identification was based on their appearance as circular, dome-shaped, dark-pigmented (black to brown), raised surface colonies displaying an autofluorescent brick-red color under long-wave ultraviolet light exposure, and testing negative for lactose fermentation activity. *P. micra* presented as Gram-positive, non-motile, anaerobic cocci exhibiting small (minute to 1.0 mm in diameter), shiny, non-hemolytic, catalase-negative, opaque white, circular, convex surface colonies. *F. nucleatum* group species were identified as long-wave ultraviolet light autofluorescent charteuse-positive, gray, iridescent colonies of Gram-negative, filamentous, spindle-shaped, non-motile rods. *S. constellatus* was defined as Gram-positive, lactose-negative, non-motile, facultative cocci demonstrating small white, opaque, circular, β-hemolytic, surface colonies with irregular edges, and positive for α-D-glucosidase enzyme activity. *C. rectus* was identified on the basis of mobility, cellular morphology, and the formation of small (1–3 mm), catalase and oxidase-negative, pale-translucent colonies appearing as either corroding, spreading, or convex variants.

*P. intermedia* was not differentiated from *P. nigrescens*, but a subset of isolates phenotypically identified as *P. intermedia/nigrescens* were confirmed to be either *P. intermedia* or *P. nigrescens* with matrix-assisted laser desorption-ionization time-of-flight (MALDI-TOF) mass spectrometry and Bruker MALDI Biotyper analytic software (Bruker Daltonics, Billerica, MA, USA), following procedures previously described [38,39]. This method was also used to confirm species identification of other selected clinical isolates of test species. Proportional patient recovery of test species per subgingival specimen was calculated as the percent recovery of colony forming units (CFU) of the species among total cultivable anaerobic viable counts as determined on non-antibiotic supplemented EBBA plates.

### 2.5. In Vitro Antibiotic Resistance Testing

Growth of test species on antibiotic-supplemented EEBA plates indicated their in vitro resistance to the evaluated antibiotic breakpoint concentration [33,35]. This direct plating method, with non-susceptible antibiotic breakpoint concentrations incorporated into primary isolation plates, has shown excellent correlation (*r*^2^ = 0.99) with the CLSI-approved agar dilution susceptibility assay for identification of antibiotic-resistant periodontal microorganisms [40]. *Bacteroides thetaiotaomicron* ATCC 29741, *Clostridium perfringens* ATCC 13124, and a multi-antibiotic-resistant clinical periodontal isolate of *F. nucleatum* were used as positive and negative quality controls for all antibiotic resistance testing on antibiotic-supplemented EBBA plates.

### 2.6. Laboratory Protocol and Study Approval

All laboratory procedures were performed following a standardized protocol by same laboratory personnel, who were masked to the clinical status and diagnosis of the sampled patients, and their inclusion into the present study. Approval for the study was provided by the Temple University Human Subjects Institutional Review Board, which determined that secondary use of normally discarded subgingival specimens not linked to the identification of any person(s) did not qualify as human subjects research requiring written patient consent since patient identities and contact information were not known, and no investigator-study patient contact, interaction, or intervention was carried out.

### 2.7. Data Analysis

For each of the evaluated red/orange complex species, the number and proportion of species-positive patients from non-antibiotic containing EBBA plates was determined, along with its mean cultivable subgingival proportional recovery and SD, as well as the number and proportion of patients positive for antibiotic-resistant strains of the species, and proportional recovery of drug-resistant strains on various antibiotic-supplemented EBBA plates. Fisher’s exact test compared among patients the presence one or more tinidazole-resistant red/orange species, as compared to the presence in patients of metronidazole, amoxicillin, doxycycline, or clindamycin-resistant test species. Total cultivable subgingival proportions of red/orange complex species per patient were determined by summing together individual species data for each patient, and then calculating total mean values across all patients, as previously described [41]. A paired t-test compared mean total cultivable subgingival proportions of red/orange complex species per patient resistant in vitro to tinidazole, as compared to mean proportions per patient resistant to either metronidazole, amoxicillin, doxycycline, or clindamycin. In vitro antibiotic-resistance data for tinidazole and metronidazole were each combined post hoc with those for amoxicillin, and compared per study patient, as previously described [33,35], to determine the number and proportion of patients with test species exhibiting joint in vitro resistance to both antibiotics at the employed breakpoint concentrations, since the combination of metronidazole plus amoxicillin is frequently employed in the treatment of human periodontitis [19]. A *p*-value of ≤0.05 was required for all tests of statistical significance. The PC-based STATA/SE 16.0 for Windows (StataCorp PL, College Station, TX, USA) 64-bit statistical software package was used in the data analysis.

## 3. Results

### 3.1. Total Cultivable Counts

Total cultivable subgingival anaerobic viable counts on EBBA plates without any added antibiotics averaged 1.7 × 10^8^ ± 1.3 × 10^8^ (SD) organisms/mL of sample (range = 2.0 × 10^7^ to 5.0 × 10^8^ organisms/mL).

### 3.2. Red/Orange Complex Species Recovery

Table 1 lists the presence and subgingival proportional recovery of evaluated red/orange complex species in subgingival biofilm specimens from 88 adults with severe periodontitis.

*P. micra* was isolated from all of the study patients, and *P. intermedia/nigrescens* and *F. nucleatum* from 90.9% and 83.0% patients, respectively, with mean subgingival proportions of these species in culture-positive patients ranging from 7.1% to 8.5%. *T. forsythia* was recovered from 53.4% of the patients, and *P. gingivalis* from 10.2% patients. Total cultivable subgingival proportions of evaluated red/orange complex species per patient averaged 24.5 ± 17.3 (SD) % on non-antibiotic containing EBBA plates.

### 3.3. In Vitro Antibiotic Resistance Testing

Table 2 lists the number and percentage of patients with red/orange complex species exhibiting in vitro resistance to the evaluated antibiotic breakpoint concentrations.

Tinidazole inhibited in vitro growth of all *P. gingivalis* isolates from 9 patients, *T. forsythia* from 47 patients, *F. nucleatum* from 73 patients, and *C. rectus* from 13 patients (Table 2). However, tinidazole failed to inhibit in vitro growth of *P. intermedia/nigrescens* from 3 (3.8%) of 80 species-positive patients, *P. micra* from 9 (10.2%) of 88 patients, and *S. constellatus* from 8 (88.9%) of 9 patients (Table 2). Metronidazole exhibited similar in vitro drug resistance patterns as tinidazole (Table 2), except for significantly less in in vitro resistance by *P. micra* clinical isolates to metronidazole as compared to tinidazole (*p* < 0.05, Fisher’s exact test). In vitro resistance to amoxicillin, doxycycline, and clindamycin by the evaluated red/orange complex species was significantly greater than was found to tinidazole or metronidazole (all *p*-values < 0.05, Fisher’s exact test). This increased resistance of red/orange complex species was particularly pronounced to clindamycin, where subgingival isolates of *T. forsythia*, *P. micra*, and *P. intermedia/nigrescens* were resistant in vitro to clindamycin among more than 50% of species-positive patients (Table 2).

Table 3 presents the prevalence among patients, and mean total cultivable subgingival proportions of red/orange complex species per patient resistant to the test antibiotics, including post-hoc combination of data for tinidazole plus amoxicillin, and metronidazole plus amoxicillin.

Patients positive with tinidazole-resistant red/orange complex species (23.9%) were significantly less prevalent than patients positive with amoxicillin (53.4%), doxycycline (61.4%), or clindamycin-resistant (77.3%) test species (all *p* values < 0.001, Fisher’s exact test), but not those with metronidazole-resistant strains (13.6%) (*p* = 0.08, Fisher’s exact test). Post-hoc combination of in vitro resistance data for tinidazole plus amoxicillin, and for metronidazole plus amoxicillin, revealed both of these antibiotic combinations able to inhibit all red/orange complex species among the study patients, except for a subset of *P. intermedia/nigrescens* clinical isolates resistant to both tinidazole and amoxicillin for 3 patients, and resistant to both metronidazole and amoxicillin for one patient.

Mean total cultivable subgingival proportional levels of tinidazole-resistant red/orange complex species per patient (2.5%) were significantly less than cultivable proportions per patient found with amoxicillin (4.7%), doxycycline (7.2%), and clindamycin (9.9%) (all *p*-values < 0.05, paired t-test), but not with metronidazole (1.0%), which were significantly lower than with tinidazole (*p* < 0.01, paired t-test) (Table 3). No significant differences were found between data combined post-hoc for tinidazole plus amoxicillin, as compared to metronidazole plus amoxicillin, relative to mean cultivable subgingival proportional levels of jointly-resistant red/orange complex species per patient (0.5 ± 3.5 (SD) % versus 0.02 ± 0.14 (SD) %, respectively; *p* = 0.195, paired t-test).

## 4. Discussion

The major finding from this study is that tinidazole performed similar to metronidazole, and markedly better than amoxicillin, doxycycline, and clindamycin, with regard to in vitro inhibition of fresh clinical isolates of red/orange complex periodontal pathogens recovered from adults with severe periodontitis. Significantly fewer patients yielded tinidazole-resistant test species, and had significantly lower cultivable subgingival proportional levels of tinidazole-resistant organisms per patient, than patients with amoxicillin, doxycycline, or clindamycin-resistant species. The prevalence of patients with one or more tinidazole-resistant red/orange complex species was not significantly different from those with metronidazole-resistant isolates, but cultivable subgingival levels of test species per patient resistant to tinidazole were significantly higher than those resistant to metronidazole. Only *P. micra*, among the evaluated red/orange complex species, exhibited significantly more in vitro resistance to tinidazole than metronidazole, even though 89.8% of *P. micra*-positive patients had all clinical isolates of the organism susceptible to 16 mg/L of tinidazole. These in vitro antibiotic resistance findings are clinically relevant to the selection of systemic antibiotics to be potentially employed in periodontitis therapy since antibiotics ineffective in vitro against major periodontal pathogens under ideal laboratory test conditions with planktonic microbial cells are unlikely to be of much therapeutic benefit against periodontal pocket microbial biofilms during in vivo administration [7].

This study also provides the first published data on in vitro susceptibility to tinidazole among subgingival *P. gingivalis*, *T. forsythia*, *P. micra* and *C. rectus* from severe periodontitis patients, and all clinical isolates of these species were found to be susceptible to clinically-attainable therapeutic concentrations of tinidazole as well as metronidazole. The low frequency of tinidazole resistance by *P. intermedia/nigrescens* from 80 patients (3.8%), and none by *F. nucleatum* from 73 patients, in the present study are in agreement with a previous report where only one of 10 periodontal *P. intermedia* isolates, and none of 10 periodontal *F. nucleatum* strains, exhibited in vitro resistance to 16 mg/L of tinidazole [31].

Neither tinidazole nor metronidazole were found to be effective in vitro against *S. constellatus*, a Gram-positive facultative cocci implicated in progressive periodontitis, and poorly susceptible to imidazole class antibiotics [33,42], including tinidazole [43]. This was expected since tinidazole has only limited antimicrobial activity against non-anaerobic bacteria [44,45,46], which do not possess ferredoxin or flavodoxin-like metabolic pathways needed for intracellular tinidazole drug reduction and formation of redox intermediate metabolites that interfere with microbial nucleic acid synthesis [27,44,47]. Thus, for severe periodontitis patients jointly colonized by high subgingival levels of both anaerobic and facultative periodontal pathogens (such as anaerobic red/orange complex species and facultative *S. constellatus* and/or *Aggregatibacter actinomycetemcomitans*), combined use of tinidazole with another antibiotic having a complementary but different range of antimicrobial activity may be indicated to broaden the antimicrobial spectrum of drug chemotherapy [7,18,33]. This was done in a clinical study of diabetes-associated periodontitis patients, where a systemic combination of tinidazole plus ampicillin was prescribed as an adjunct to conventional mechanical-surgical periodontal therapy [48]. The present in vitro study findings show that tinidazole plus amoxicillin would provide similar broad-spectrum antimicrobial activity as metronidazole plus amoxicillin, a drug combination frequently employed in human periodontal disease treatment [19]. Tinidazole additionally has been shown to exhibit antimicrobial synergism in vitro in combination with either amoxicillin/clavulanic acid, levofloxacin, or clindamycin against a mixed bacterial inoculum of periodontal origin [32]. Pharmacokinetic studies are needed to determine the best dosing schedules for such possible combined antibiotic regimens in light of differing half-life values for tinidazole and other non-imidazole antibiotics, such as amoxicillin [27].

Since tinidazole has a 12–14 hour serum half-life, which is approximately double that found with metronidazole [49], providing mean gingival crevicular fluid concentrations of 13 mg/L of tinidazole at 24 hours after a single 2-gram oral dose [23], tinidazole requires less frequent daily patient dosing than metronidazole, and thereby offers an advantage over metronidazole relative to attaining patient compliance with prescribed drug consumption frequency. As a result of these more favorable pharmacokinetic properties permitting a more convenient once-a-day oral dose schedule for tinidazole, its similar array of potential side effects and drug interactions as metronidazole [26], and its similar antimicrobial activity against red/orange complex periodontal pathogens as metronidazole, as documented in the present study, tinidazole should be considered in place of metronidazole for systemic periodontitis drug therapy, particularly when patient compliance with a multiple dose per day drug regimen is anticipated to be poor or difficult to attain. This would be in accord with findings that patient compliance with drug dosing improves as the prescribed dose frequency decreases [24], and recommendations that medications be favored that have the lowest daily prescribed dose frequency [24]. However, systemic use of tinidazole, as well as with all other antibiotics in periodontal therapy, should be selective in nature and reserved mostly for periodontitis patients having a poor clinical outcome, and/or elevated persistence of key periodontal pathogens, after completion of conventional mechanical periodontal treatment [18,50]. Periodontal microbiology laboratory analysis may assist dental professionals in reducing the risk of therapeutic failure inherent with empirically prescribed systemic periodontal antibiotic therapy [51] by identifying periodontal pathogens with either predictable antibiotic susceptibility patterns or which display in vitro resistance to antibiotics under consideration for in vivo use [7]. This approach will help limit overall patient exposure to antibiotics, reduce development of adversely altered microbial populations at non-oral body sites, and lower the risk of increased antibiotic resistance in the human microbiome. Additional in vitro and clinical research is needed to further evaluate the potential use and efficacy of tinidazole in treatment of human periodontal disease.

## 5. Conclusions

In vitro resistance to tinidazole was similar to metronidazole among fresh clinical isolates of red/orange complex periodontal pathogens from severe periodontitis patients, and markedly less than to amoxicillin, doxycycline, or clindamycin. As a result of its similar antimicrobial spectrum against red/orange complex periodontal pathogens, and more convenient once-a-day oral drug dosing, tinidazole should be considered in place of metronidazole for systemic periodontitis drug therapy when it is clinically and/or microbiologically indicated, particularly when patient compliance with a multiple dose per day drug regimen is anticipated to be poor or difficult to attain.

## Figures and Tables

**Table 1:** Red/orange complex species recovered from study patients.

Test Species	No. (%) of Positive Patients	Mean % Recovery in Species- Positive Patients (SD) *	Range %
**Red Complex Species:**			
*P. gingivalis*	9 (10.2)	10.1 (6.6)	1.0–21.0
*T. forsythia*	47 (53.4)	2.3 (1.9)	0.2–10.5
**Orange Complex Species:**			
*P. intermedia/nigrescens*	80 (90.9)	7.1 (9.9)	0.1–46.7
*P. micra*	88 (100)	7.9 (6.3)	0.2–40.0
*F. nucleatum*	73 (83.0)	8.5 (6.4)	0.7–32.6
*S. constellatus*	9 (10.2)	7.4 (4.5)	2.4–13.3
*C. rectus*	13 (40.9)	0.2 (0.2)	0.1–1.0

* calculated as the average percent CFU recovery of the species among total cultivable anaerobic viable counts as determined on non-antibiotic supplemented EBBA plates among patients culture-positive for the test species.

**Table 2:** Patients with antibiotic-resistant red/orange complex species (antibiotic concentration).

Test Species		TIN (16 mg/L)	MET (16 mg/L)	AMOX (8 mg/L)	DOXY (4 mg/L)	CLIN (4 mg/L)
**Red Complex Species:**						
*P. gingivalis*	N *	0	0	1 (11.1)	0	2 (22.2)
	% †	0	0	7.6	0	5.5 ± 6.4
*T. forsythia*	N	0	0	14 (29.8)	5 (10.6)	25 (53.2)
	%	0	0	2.7 ± 1.4	3.2 ± 1.7	2.4 ± 1.6
**Orange Complex Species:**						
*P. intermedia/nigrescens*	N	3 (3.8)	1 (1.3)	40 (50.0)	38 (47.5)	47 (58.8)
	%	14.7 ± 14.6	2.8	9.1 ± 10.4	8.2 ± 11.5	7.3 ± 9.7
*P. micra*	N	9 (10.2)	2 (2.3)	2 (2.3)	34 (38.6)	48 (54.6)
	%	10.1 ± 5.8	9.3 ± 9.5	4.2 ± 1.3	8.4 ± 5.8	8.2 ± 6.7
*F. nucleatum*	N	0	0	2 (2.7)	3 (4.1)	0
	%	0	0	7.9 ± 2.9	6.5 ± 3.1	0
*S. constellatus*	N	8 (88.9)	9 (100)	0	2 (22.2)	0
	%	7.4 ± 4.5	7.4 ± 4.5	0	11.3 ± 2.9	0
*C. rectus*	N	0	0	0	0	0
	%	0	0	0	0	0

TIN = tinidazole. MET = metronidazole. AMOX = amoxicillin. DOXY = doxycycline. CLIN = clindamycin. * Number (%) of patients with antibiotic-resistant strains of species among total species-positive patients. † Mean ± SD percentage cultivable recovery of antibiotic-resistant strains of species in positive patients.

**Table 3:** Presence and levels of antibiotic-resistant red/orange complex species per patient.

Antibiotic (Breakpoint Concentration)	No. (%) Patients Positive with Drug-Resistant Red/Orange Complex Species	Mean (SD) Total Cultivable Subgingival Proportions of Drug-Resistant Red/Orange Complex Species Per Patient
clindamycin (4 mg/L)	68 (77.3)	9.9 (12.2)
doxycycline (4 mg/L)	54 (61.4)	7.2 (10.7)
amoxicillin (8 mg/L)	47 (53.4)	4.7 (8.7)
tinidazole (16 mg/L)	21 (23.9)	2.5 (5.6)
metronidazole (16 mg/L)	12 (13.6)	1.0 (3.1)
tinidazole (16 mg/L) plus amoxicillin (8 mg/L) *	3 (3.8)	0.5 (3.5)
metronidazole (16 mg/L) plus amoxicillin (8 mg/L) *	1 (1.3)	0.03 (0.3)

* Post-hoc combination of in vitro resistance data for both antibiotics.
